# Lessons for the HIV response from structural innovations catalysed by COVID-19

**DOI:** 10.1136/bmjgh-2022-010854

**Published:** 2022-11-29

**Authors:** Michael Isbell, Linda-Gail Bekker, Beatriz Grinsztejn, Jennifer Kates, Adeeba Kamarulzaman, Sharon R Lewin, Kenneth Ngure, Nittaya Phanuphak, Anton Pozniak, Anna Grimsrud

**Affiliations:** 1 Consultant, New York, New York, USA; 2 The Desmond Tutu HIV Centre, University of Cape Town, Rondebosch, South Africa; 3 Evandro Chagas National Institute of Infectious Diseases, Oswaldo Cruz Foundation (FIOCRUZ), Rio de Janeiro, Brazil; 4 Henry J Kaiser Family Foundation, Washington, District of Columbia, USA; 5 University of Malaya, Kuala Lumpur, Malaysia; 6 Department of Medicine, University of Malaya, Kuala Lumpur, Malaysia; 7 Department of Infectious Diseases, The Peter Doherty Institute for Infection and Immunity Melbourne, University of Melbourne, Melbourne, Florida, Australia; 8 The Peter Doherty Institute for Infection and Immunity, Royal Melbourne Hospital, Melbourne, Florida, Australia; 9 School of Public Health, Jomo Kenyatta University of Agriculture and Technology, Nairobi, Kenya; 10 Institute of HIV Research and Innovation, Bangkok, Thailand; 11 Department of HIV Medicine, Chelsea & Westminster Hospital NHS Foundation Trust, London, UK; 12 Department Clinical Research, London School of Hygiene & Tropical Medicine, London, UK; 13 HIV Programmes & Advocacy, International AIDS Society, Cape Town, South Africa

**Keywords:** HIV, COVID-19, Public Health

**Summary box**

- While much has been written on how the HIV response laid the groundwork for the COVID-19 response, this analysis examined structural innovations used to respond to COVID-19 and assessed their potential utility for the future of the HIV response.
- Our analysis indicates certain COVID-19-related innovations are clearly relevant and have potential value for the HIV response moving forward.
- Public–private partnerships that catalysed the unprecedented, rapid development of COVID-19 vaccines and the numerous innovations in the design and conduct of clinical trials that made them more efficient and reduced burdens on trial participants have important lessons for the HIV response.
- Experience with COVID-19 diagnostics underscores the need to bring underused HIV point-of-care diagnostic technologies to scale and increase access to HIV viral load monitoring.
- COVID-19 also highlighted the importance of enhancing the timeliness and strategic use of data for HIV-related decision-making and programmatic adaptations.
- Other COVID-19-related innovations, such as the Access to COVID-19 Tools Accelerator and COVID-19 Vaccines Global Access initiative, are less immediately applicable to HIV, in part due to the success of the HIV response in developing analogous mechanisms.
- The failure of any manufacturer of a COVID-19 vaccine to use the voluntary licensing mechanism of the Medicines Patent Pool has slowed vaccination uptake and provides an important lesson for what the HIV response ought not to do for future HIV prevention, treatment and cure breakthroughs.
- The global community’s failure to ensure equitable access to COVID-19 vaccines and treatments highlights the enduring importance of principles of global solidarity and shared responsibility in addressing global health challenges.

## Introduction

Substantial evidence indicates HIV investments have yielded important benefits for the world’s response to COVID-19.^1 2^ HIV research provided the foundation for COVID-19 vaccines^3 4^; health surveillance systems in southern Africa, built in part through HIV funding, provided early warnings regarding SARS-CoV-2 variants^5^; and HIV service and clinical trial sites, diagnostic platforms and community systems rapidly pivoted to address both HIV and COVID-19.^6^

While these synergies underscore the value of investments in the HIV response, it is also clear that the HIV response requires new energy and commitment. In 2021, 1.5 million people were newly infected with HIV, with the global pace of decline in new infections in 2021, the smallest in 5 years.^7^ Essential financing for the HIV response has flattened at levels markedly below what is needed to end AIDS as a public health threat by 2030.^7^

Given the urgency of rejuvenating the response to HIV, we sought to assess whether structural innovations used to respond to COVID-19 might hold promise for accelerating progress towards ending AIDS. We undertook an extensive review of the peer-reviewed and grey literature on COVID-19-related innovations and drew from our respective professional backgrounds in the HIV field to identify approaches that might be applicable to the HIV response moving forward. Our literature review was complemented by interviews with key informants who had experience working within or liaising with the mechanisms established to respond to COVID-19.

## COVID-19 innovations of potential value to the HIV response

**Figure 1:**
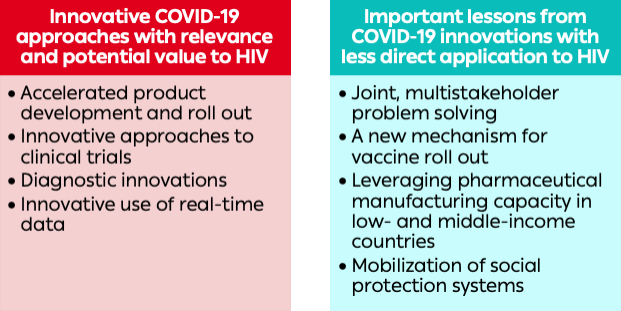
Structural innovations used to respond to COVID-19 with relevance and potential value and less direct application to the HIV response.

In response to the COVID-19 pandemic, diverse stakeholders adopted new ways of doing business, in some cases by rapidly implementing innovations that had long been proposed but seldom brought to scale. Several of these innovations have clear relevance to HIV (figure 1).

### Accelerated product development and roll out

Before COVID-19, the fastest time from the laboratory to introduction of a new vaccine was 4 years.^8^ During the COVID-19 pandemic, clinical development of the earliest vaccines took a mere 7 months.^9 10^

While a number of factors contributed to the unprecedented speed of COVID-19 vaccine development, extraordinary investments by governments in both ‘push’ and ‘pull’ mechanisms to drive innovation played an important role. Push mechanisms involve direct investments in applied research and development, while pull mechanisms (typically in the form of advance market commitments) aim to minimise delays associated with perceived financial risks to the vaccine maker. Advance market commitments, for example, worked to accelerate the roll out of pneumococcal vaccines,^11^ which are now being used in more than 60 countries to prevent deaths in young children.^12^

Through a combination of push and pull mechanisms, public investments were pivotal in the development and roll out of COVID-19 vaccines.^13 14^ The largest of these public programmes—the US Government’s Operation Warp Speed, subsequently renamed COVID-19 Countermeasures Acceleration Group—yielded multiple validated vaccines through investments in research and development and advance market purchases before any vaccine had completed clinical trials, at a cost of US$ 29.8 billion.^10 14^ While Operation Warp Speed was remarkably successful in spurring the development of new vaccines, the USA proved less successful than many other countries in rolling out vaccines,^15^ underscoring the importance of paying as much attention to delivery issues as to the scientific challenges of vaccine development.

While the public–private partnership model of Operation Warp Speed and other similar push–pull initiatives is unlikely to have immediate application in the HIV field, as no HIV vaccine appropriate for broad scale-up is currently on the horizon, they offer important models for the future. There is considerable excitement in the HIV vaccine field over potential applications of the mRNA technologies that have proven transformative in the case of COVID-19 vaccination.^16^ Should early-phase trials point strongly toward the effectiveness of one or more HIV vaccine candidates, advance market commitments, by guaranteeing a market, encourage makers to manufacture sufficient quantities to begin roll out as soon as a vaccine receives regulatory approval, shaving months or even years off the timeline for broad-based introduction.

### Innovative approaches to clinical trials

COVID-19 demonstrated that clinical trials of novel vaccines and therapeutics can be conducted much faster than in the past. According to a comprehensive analysis, operational efficiencies in COVID-19 vaccine trials shaved off at least 70% of the time that would have been required to yield trial results had pre-COVID-19 research approaches been followed.^9^

Some of the innovations that helped expedite evaluation of COVID-19 vaccines and therapeutics—such as combining trial phases I, II and III or running them in parallel—are so costly that they may not be feasible in the case of an HIV vaccine, treatment or curative regimen unless and until early clinical trial data strongly suggest that one or more candidates are likely to be highly effective and feasible for rapid roll out. However, other efficiency-promoting innovations used during COVID-19 appear more clearly applicable to HIV-related research. These include contracting efficiencies, decentralised trial structure, digital patient support, real-time data processing, comprehensive organisational alignment of the research team and changes in behavioural and business norms, including new approaches to multistakeholder partnerships.^9^ Adaptive trial designs, including the pooling of the placebo group and use of a master protocol for researchers testing different compounds, as well as virtual recruiting and monitoring, have also contributed to clinical trial efficiencies of both vaccines and treatments in the context of COVID-19 (and are now being applied to monkeypox-related research).^17–19^ Many of these trial innovations, which have the added benefit of reducing burdens associated with trial participation, may warrant mainstreaming across HIV clinical research.

### Diagnostic innovations

The decades-long evolution in HIV testing continues, with an early focus on facility-based antibody testing giving way to a proliferating array of rapid point-of-care and home testing platforms.^20^ In the case of COVID-19, the evolution in diagnostics happened much more swiftly. Increasingly, COVID-19 testing is being performed at home through rapid antigen tests.^21^ At-home COVID-19 testing satisfies the preferences of many people, provides immediate and useful health information and avoids the need to wait (in some settings for several days) for test results. In settings where self-reporting of positive test results is uncommon, the growth in at-home testing has made surveillance through lab reporting of COVID-19 test results less reliable.

The WHO has endorsed HIV self-testing^22^ and encouraged countries to remove policy barriers to scale-up of this tool.^23^ A growing number of countries has endorsed HIV self-testing. Other user-friendly innovations, including point-of-care diagnostics for early infant diagnosis and HIV viral load monitoring, have the potential to overcome HIV diagnostic bottlenecks.^24^

COVID-19 also led to a substantial increase in PCR capabilities in low-income and middle-income countries. PCR tests remain either laboratory based or as point-of-care technologies, but are critical for monitoring HIV viral load. Historically, many countries have lacked access to PCR technology, but this has changed dramatically as a result of COVID-19. Furthermore, home-based viral load testing is an urgently needed technology, specifically for monitoring of HIV cure interventions. The explosion of innovations in COVID-19 diagnostics may well precipitate the development of home-based viral load monitoring for HIV.

Leveraging lessons learnt from COVID-19, the HIV response now needs to invest in strategic actions to scale up these diagnostic innovations, which to date have achieved insufficient coverage. While the number of self-tests distributed is rising, self-testers represent only a small fraction of people who would benefit from HIV testing services.^25^ Similarly, one in three infants exposed to HIV in 2020 received no virologic testing within the first 2 months of life, highlighting coverage deficits for both point-of-care and traditional diagnostic testing through centralised laboratories.^26^ In the case of HIV self-testing, a 2022 agreement for pricing of a leading HIV self-test at 50% below prevailing market rates offers hopes for expanded access to this HIV diagnostic tool.^27^ Just as COVID-19 home-based testing prioritised the needs of the individual, scale-up of HIV home testing will be accelerated by further demedicalizing HIV self-testing.

### Innovative use of real-time data

COVID-19 transformed the use of health data for impact. User-friendly dashboards, updated daily, have enabled rapid public health interventions,^28^ ongoing tracking of hospital and intensive care capacity has supported health system planning,^29^ wastewater surveillance has provided early evidence of localised increases in SARS-CoV-2 incidence,^30^ tracking of mobile phone data has provided evidence of the effectiveness of COVID-19 lockdowns^31^ and molecular epidemiology has accelerated the identification of novel variants and phenotypic characteristics.^32^

The HIV response has generated a host of innovative approaches to the collection and strategic use of data.^33^ However, where the HIV response has lagged the COVID-19 response is in the use of real-time data. While the need for speedy access to data is more pressing for COVID-19 than for HIV, accelerating the availability of HIV-related strategic information could still have important benefits, enabling more rapid identification of gaps and bottlenecks and swifter adaptation of policy and programmatic approaches. The ability to collect real-time data for public health impact requires an integrated system built on electronic record-keeping and data dissemination,^34^ such as in the UK, where centralised data systems and data triangulation methods served as the ‘lifeblood’ of public health decision-making during COVID-19.^35^

Integrated, real-time data systems could enable more sophisticated, high-impact data collection and analysis for the HIV response. For example, integrated data systems could theoretically track pre-exposure prophylaxis (PrEP) efficacy in real-time by linking data on PrEP utilisation with reported cases of new HIV diagnosis. Although real-time molecular epidemiology has the potential to speed the identification and response to HIV transmission clusters and outbreaks,^36^ its use in the context of HIV involves ethical issues that must be addressed, taking into account the social and legal vulnerabilities of populations most heavily affected, which make contact tracing and public health interventions more fraught and complicated than they were in the case of COVID-19.^30^

## Important lessons from COVID-19 innovations with less direct application to HIV

Other COVID-19-related innovations are less immediately applicable to HIV, but still offer important lessons that the HIV response should heed (figure 1).

### Joint, multistakeholder problem-solving

The Access to COVID-19 Tools (ACT) Accelerator has brought together governments, global health initiatives (including the Global Fund), civil society and the private sector for planning, problem-solving, innovation, advocacy and resource mobilisation around four pillars of COVID-19-related work—diagnostics, treatments, vaccines and health systems strengthening.^37^ While the ACT Accelerator is an interesting collaborative model that has been found to have an additive effect,^38^ its value as a model for the HIV response is questionable, as the HIV response has already given rise to analogous, if less expansive, platforms for collective problem-solving, such as the Global HIV Vaccine Enterprise and a series of collaborative platforms addressing the paediatric HIV agenda.

### A new mechanism for vaccine roll out

The most prominent component of the ACT Accelerator, the COVID-19 Vaccines Global Access (COVAX) initiative, is also unlikely to serve as a meaningful model for eventual roll out of future HIV prevention, treatment and cure breakthroughs, if only because COVAX fell badly short of its goals.^38^ The HIV response has already developed commodity procurement and supply management mechanisms, as well as facilities and community systems to reach the adolescents and adults who will likely be targeted by early HIV vaccination campaigns or further HIV treatment innovations. However, the failure of lessons learnt from HIV to ensure rapid, equitable access to COVID-19 vaccines and treatments poses questions regarding the confidence we can have in the ability to roll out future HIV biomedical innovations. Mechanisms to ensure equitable access to essential health technologies remains work in progress, underscoring the need for further efforts by the global community to build systems for rapid roll out.

The ability of COVAX and the broader ACT Accelerator to drive rapid, effective action suffered as a result of the global community’s failure to mobilise the resources needed to ensure a globally equitable response to COVID-19.^39^ This should be of deep concern for the HIV response, which is already experiencing a reduction in international and domestic investments, leaving the response at least US$ 8 billion short of amounts needed to end AIDS by 2030.^7^ More effectively making the case for the value of HIV investments, identifying new sources of financing and galvanising investments whose benefits extend beyond individual health silos are key priorities for the HIV response.

### Leveraging pharmaceutical manufacturing capacity in low-income and middle-income countries

The COVID-19 response has struggled to enable and fully leverage pharmaceutical manufacturing capacity in all regions. Although these struggles can be traced to several factors, one important one is the refusal of the manufacturers of COVID-19 vaccines to use the access-promoting voluntary licensing scheme of the Medicines Patent Pool (MPP). As of July 2022, no COVID-19 vaccine was covered by a voluntary license. In June 2022, the World Trade Organization approved a temporary waiver of intellectual property protections for COVID-19 vaccines, but the deal was reached long after the point when the manufacture of vaccines in low-income and middle-income countries could have made a marked difference in vaccination uptake.^40^ Ensuring timely voluntary licensing, supported by the full transfer of know-how and technology, will be essential for rapid introduction of future HIV prevention, treatment and curative breakthroughs.

To facilitate technology transfer for the manufacture of COVID-19 vaccines in low-income and middle-income countries, WHO, MPP and ACT Accelerator established the mRNA vaccine technology transfer hub.^41^ The outcome of this technology transfer initiative is of potential significance for the future of the HIV response, as the hub aims not only to galvanise manufacturing capacity for COVID-19 but also HIV and other communicable diseases.^42^

### Mobilisation of social protection systems

Social protection systems aim to help poor and vulnerable people withstand crises, through such means as job programmes, cash transfers, food assistance and investments in training and education.^43^ One notable feature of the COVID-19 response was the rapid mobilisation of social protection systems to mitigate the socioeconomic effects of the COVID-19 pandemic.^44^ Global spending on social protection nearly tripled during the COVID-19 pandemic, with increased donor investments enabling substantial social protection spending in low-income and middle-income countries.^45^ Although future stages of the HIV response are unlikely to elicit the kinds of whole-of-society responses prompted by COVID-19, there are growing calls to better mobilise social protection systems to reduce HIV vulnerability and enhance service utilisation.^46^ Building social protection expertise and capacity within HIV systems, especially among communities, is an important step towards effectively leveraging social protection systems for the HIV response.

## Conclusion

Several innovations catalysed by COVID-19 warrant focused attention as innovations to be applied or adapted for the HIV response. These include the combination of push and pull mechanisms to accelerate product development, innovative trial designs coupled with expedited regulatory review, rapid dissemination of and enhanced access to breaking biomedical information, diagnostic innovations, enhanced use of real-time data and the energetic and innovative engagement of non-health sectors in the pandemic response. Other COVID-19-related innovations appear less clearly applicable to the HIV response moving forward, although their experiences offer useful lessons for the HIV response. Among these experiences are difficulties in mobilising sufficient resources, engaging the global South and civil society and overcoming barriers posed by intellectual property rules.

The COVID-19 and HIV responses share a critical characteristic. While both pandemics galvanised global action and achieved important gains, the response to each has been undermined by inadequate attention to persistent inequalities and disparities, including many that stem from the unbalanced distribution of wealth embedded in the international economic order. A shared lesson from pandemic responses over the past half-century is that sustainable success demands attention to equity in access and outcomes in every aspect of the response.

## Data Availability

No datasets were generated or analyzed during the current study.
